# High midday temperature stress has stronger effects on biomass than on photosynthesis: A mesocosm experiment on four tropical seagrass species

**DOI:** 10.1002/ece3.3952

**Published:** 2018-04-10

**Authors:** Rushingisha George, Martin Gullström, Mwita M. Mangora, Matern S. P. Mtolera, Mats Björk

**Affiliations:** ^1^ Seagrass Ecology and Physiology Research Group Department of Ecology, Environment and Plant Sciences Stockholm University Stockholm Sweden; ^2^ Tanzania Fisheries Research Institute (TAFIRI) Dar es Salaam Tanzania; ^3^ Institute of Marine Sciences (IMS) Zanzibar Tanzania

**Keywords:** biomass loss, climate change, photosynthetic performance, tropical seagrass, Western Indian Ocean

## Abstract

The effect of repeated midday temperature stress on the photosynthetic performance and biomass production of seagrass was studied in a mesocosm setup with four common tropical species, including *Thalassia hemprichii*,* Cymodocea serrulata*,* Enhalus acoroides*, and *Thalassodendron ciliatum*. To mimic natural conditions during low tides, the plants were exposed to temperature spikes of different maximal temperatures, that is, ambient (29–33°C), 34, 36, 40, and 45°C, during three midday hours for seven consecutive days. At temperatures of up to 36°C, all species could maintain full photosynthetic rates (measured as the electron transport rate, ETR) throughout the experiment without displaying any obvious photosynthetic stress responses (measured as declining maximal quantum yield, Fv/Fm). All species except *T. ciliatum* could also withstand 40°C, and only at 45°C did all species display significantly lower photosynthetic rates and declining Fv/Fm. Biomass estimation, however, revealed a different pattern, where significant losses of both above‐ and belowground seagrass biomass occurred in all species at both 40 and 45°C (except for *C. serrulata* in the 40°C treatment). Biomass losses were clearly higher in the shoots than in the belowground root–rhizome complex. The findings indicate that, although tropical seagrasses presently can cope with high midday temperature stress, a few degrees increase in maximum daily temperature could cause significant losses in seagrass biomass and productivity.

## 1 INTRODUCTION

Tidal regimes strongly influence the productivity of coastal plant systems (Bridges & McMillan, 1986; Burdick, Dionne, Boumans, & Short, 1996; Koch & Beer, 1996). In shallow seagrass habitats, high temperatures and elevated insolation frequently occur, and during low tides, extreme temperature spikes are common (Campbell, McKenzie, & Kerville, 2006; Collier & Waycott, 2014). This can greatly influence both the photosynthetic performance and above‐ and belowground biomass of many seagrasses (Björk, Short, Mcleod, & Beer, 2008; Lee, Park, & Kim, 2007). How seagrasses respond to high temperatures depend on the duration, severity, and frequency of exposure (i.e., exposure history) as well as on species’ characteristics and interactions with other environmental factors (Bulthuis, 1987; Collier & Waycott, 2014; Hurd, Harrison, Bischof, & Lobban, 2014; Lee et al., 2007). Plant productivity in general is largely governed by temperature (Berry & Raison, 1981), and for seagrasses (and other marine plants) living in the most shallow waters, the magnitude of temperature variability will to a great extent control the productivity and growth (Lee et al., 2007). Generally, photosynthesis increases with elevated temperature up to a photosynthetic optimum. Beyond this point, however, photosynthesis may decline due to a complex set of factors (Sage & Kubien, 2007), such as enzyme denaturation (Staehr & Borum, 2011), damage of the electron transport chain, and impaired photochemical activity induced by membrane injury and sulfide intrusion (Lee et al., 2007; Murata, Takahashi, Nishiyama, & Allakhverdiev, 2007; Wahid, Gelani, Ashraf, & Foolad, 2007). High temperatures also increase plant respiration (Jordà, Marbà, & Duarte, 2012; Lee et al., 2007; Pedersen, Colmer, Borum, Zavala‐Perez, & Kendrick, 2016), which influences the productivity of the seagrass. The standing crop of seagrass plants is the sum of productivity and biomass degradation, factors that will both be affected by temperature and light conditions. There is a lot of information on effects of temperature on photosynthesis of seagrasses (Campbell et al., 2006; Collier, Uthicke, & Waycott, 2011; Collier & Waycott, 2014; Pedersen et al., 2016), while little is known regarding simultaneous loss of biomass at extreme temperatures, especially comparing multiple seagrass species (but see e.g., Collier & Waycott, 2014).

In the Western Indian Ocean (WIO), shallow‐water environments are largely inhabited by seagrasses, forming extensive lush meadows (Aleem, 1984; Gullström et al., 2002) providing important ecosystem services such as the functioning as habitat and nursery ground for fish and invertebrates (de la Torre‐Castro & Rönnbäck, 2004) and the sequestration and storage of coastal “blue” carbon (Gullström et al., 2017). This region encompasses a high diversity of seagrass species of which many inhabit the upper subtidal and lower intertidal. At low tide (especially at spring tide) during daytime, a high irradiance combined with a low water level may cause the water to be heated by several degrees over periods of 3–4 hr (Collier & Waycott, 2014; Pedersen et al., 2016). Such high temperature spikes could cause heat stress to the seagrasses, as they are living in an environment with temperatures regularly exceeding optimal levels of tolerance (Campbell et al., 2006; Collier & Waycott, 2014; Pedersen et al., 2016). When the water temperature increases above optimal levels, photosynthesis will decline rapidly, and furthermore, the optimal temperature for photosynthesis may also change with the irradiance level (Lee et al., 2007). Thus, the projected increase in sea surface temperature under a global warming scenario, which is linked to an increase in the frequency and severity of temperature spike events (Pachauri et al., 2014), would aggravate heat stress upon the seagrasses. Seagrasses have been found to be capable of a certain physiological adaptation to high temperatures (Drew, 1979; Evans, Webb, & Penhale, 1986; Zimmerman, Smith, & Alberte, 1989), but periods of high temperature have been seen to cause rapid and large losses in plant biomass (Lee, Park, & Kim, 2005). Thus, such a future scenario could threaten the survival of intertidal seagrasses in the WIO region and other tropical shallow‐water environments. An improved understanding of temperature responses in nearshore tropical seagrasses will yield better predictions of global warming impacts on the productivity, distribution patterns, and carbon dynamics of coastal habitats.

In this study, we applied a mesocosm setup aiming to investigate the effects of midday temperature stress, repeated daily for 7 days and at five different temperature treatment levels, on the photosynthetic performance and biomass of four habitat‐building tropical seagrasses. We explicitly tested the hypotheses that: (1) photosynthetic performance is influenced at similar temperature stress levels as above‐ and belowground biomass loss, (2) there are species‐specific threshold levels where photosynthetic performance and biomass are reduced, and (3) the effect of midday temperature stress on photosynthetic performance will increase with days of repeated stress.

## 2 MATERIALS AND METHODS

### 2.1 Plant material

Intact sods (0.25 × 0.25 m) of four seagrass species—*Thalassia hemprichii* (Ehrenberg) Ascherson, *Cymodocea serrulata* (R. Brown) Ascherson & Magnus, *Enhalus acoroides* (Linnaeus f.) Royle, and *Thalassodendron ciliatum* (Forsskål) den Hartog—all commonly distributed in the WIO region (Gullström et al., 2002), were collected at four separate occasions (3 days before the start of an experimental run) from February to March 2014 at the Mbweni area, Unguja Island (Zanzibar), Tanzania (6°21′S, 39°20′E). In the collection site, the four seagrass species grow in the upper subtidal, at a similar depth range and are affected by similar wave exposure level. Before the experiment, we estimated species‐specific seagrass shoot densities in the collection area, which were 880 ± 25 shoots m^−2^ (mean ± *SE*) for *T. hemprichii*, 576 ± 15 shoots m^−2^ for *C. serrulata*, 112 ± 9 shoots m^−2^ for *E. acoroides* and 288 ± 12 shoots m^−2^ for *T. ciliatum*. Seagrasses were collected using a 0.25 × 0.25 m and 0.3 m deep stainless steel corer, which was pushed into the sediment so that seagrass sods of a particular seagrass species could be carefully lifted out and still reflect the shoot density of the collection area. The sods were subsequently transported to the experimental site (at Buyu, a facility of the Institute of Marine Sciences, University of Dar es Salaam; 6°26′S, 39°23′E) located about 7 km from the collection site. Seagrass sods of all four species were deployed in each of five 100‐L white plastic containers, with the sods of the different species being arranged in separate sections of each container. The five containers, each with sods of the four seagrass species, were then placed separately in five larger, 400‐L white plastic containers containing seawater (below the rim of the smaller container), for buffering against undesirable temperature fluctuations. The 100‐L containers were filled with 80 L of seawater and bubbled with air from electrical pumps to facilitate water mixing. Each such container setup was exposed to a different temperature treatment, as given below (see “Experimental setup”). Before the start of an experimental run, the plants were allowed to acclimatize for 3 days.

### 2.2 Experimental setup

**Figure 1:**
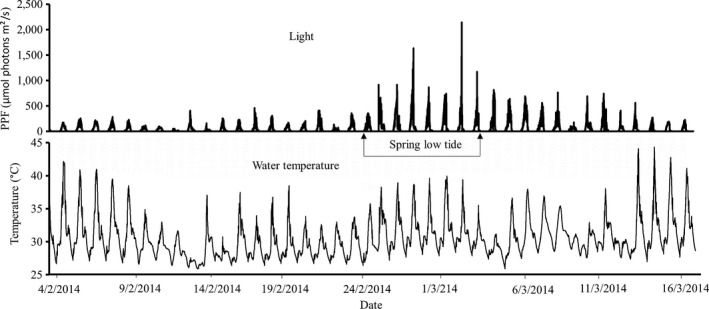
In situ temperature and light logged from February to March at the Mbweni seagrass meadow from where experimental plants were collected. Note that spring low tide conditions are indicated in the graph (to be compared to experimental conditions; see Figure 2)

**Figure 2:**
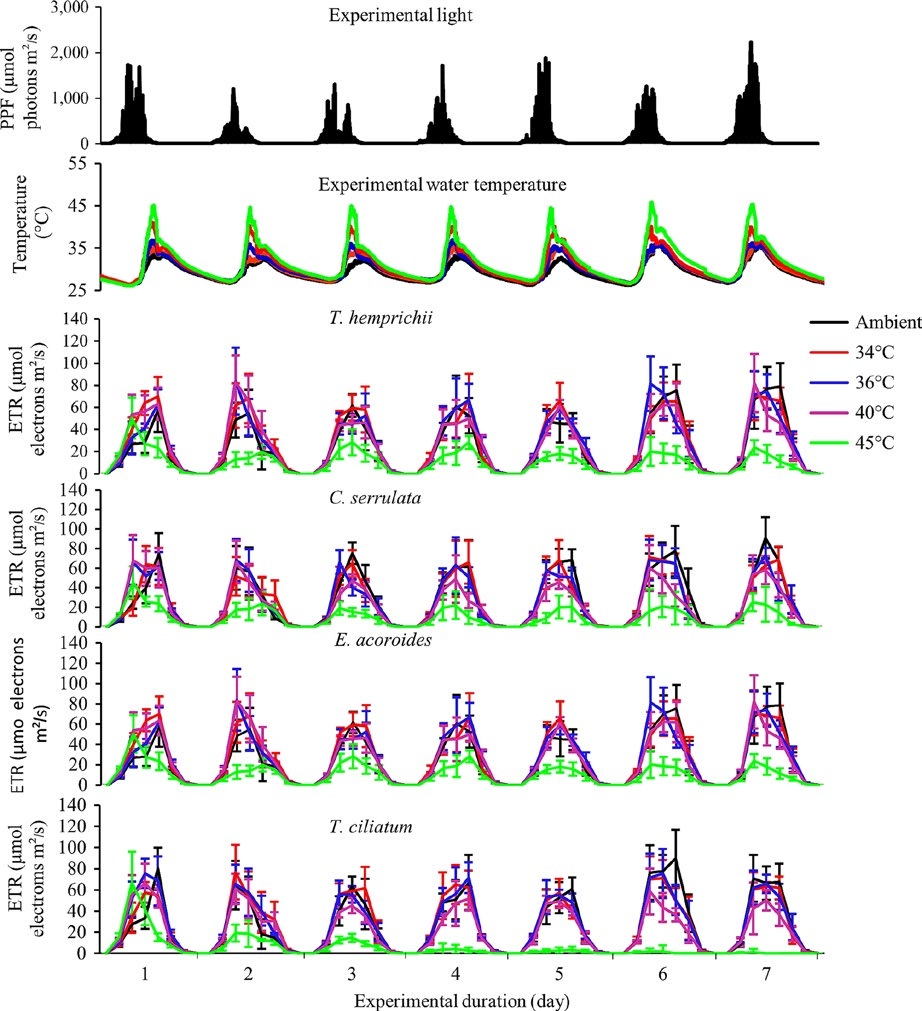
Electron transport rates (ETRs) measured in the four studied seagrass species exposed to midday temperature stress during ambient light conditions. Values are means ± *SE* (*n* = 4)

The experiment was performed outdoors under ambient light conditions (Figure 1) from the 1st of February to the 24th of March 2014, during the northeast monsoon, when seagrasses in the region normally experience stable conditions with relatively high average temperatures. Due to logistical constraints, that is, the time it took to perform measurements with available equipment, the replication of the experiment could not be performed simultaneously; instead, the full setup was repeated four times (approximately every second week) with new plant material and water. The weather conditions were similar throughout the four experimental runs (with no extreme weather events), thus rendering the four experimental runs to be comparable while still catching natural variability in, for example, light and temperature. In each experimental run, seagrass plants were exposed to five different temperature treatments: ambient (29–33°C, average: 31°C), 34, 36, 40, and 45°C. The heat stress was applied for three midday hours (10:00–13:00, to mimic the low tide exposure; cf. Figure 2) for seven consecutive days, by warming the water with submersible thermostatic heaters until the targeted temperatures were reached (after up to 2 hr). After the heat stress period, approximately 75% of the seawater in the experiment containers were gradually drained and replaced with new seawater of ambient temperature in order to lower the experimental temperatures to ambient levels (to mimic a returning high tide and also to avoid nutrient limitation). The temperature levels of the experimental temperature treatments were determined based on pilot measurements (data not shown) and previous experimental work from tropical shallow waters (Campbell et al., 2006; Collier & Waycott, 2014) as well as considering predicted future temperatures in 2,100 under a global warming scenario (Pachauri et al., 2014). The ambient containers were also partially drained, with approximately 75% being removed and refilled once per experimental run (on the third day).

### 2.3 Measurements of temperature and light

To assess natural fluctuations of water temperature and light, combined temperature and light loggers (HOBO Pendant Temp/Light Logger 8K; Onset, Bourne, MA, USA) were attached among seagrass shoots (at approximately 10 cm above the ground) in situ during February and March in the area where the seagrasses were collected. The loggers recorded water temperature (°C) and light (lux) every 30 min. Loggers were installed in a similar way in each treatment of the experimental setup. Data were retrieved after 21 days (for field loggers) and after 7 days of each experimental run (for experimental setup loggers). The light measurements recorded by the loggers were converted to μmol photons m^−2^ s^−1^ by calibrating the light logger against a PAR sensor (Model IL 1400A photometer; International Light Technologies, Peabody, MA, USA).

### 2.4 Determination of physiological effects of heat stress

The physiological effects of heat stress were assessed from chlorophyll fluorescence measurements of the maximal quantum yield (Fv/Fm, on dark‐adapted samples) and effective quantum yield (ΔF/Fm’, on ambient light‐adapted samples) of photosystem II using a pulse amplitude modulated (PAM) fluorometer (Diving PAM; Walz, Effeltrich, Germany). The tip of the instrument's optical fiber was placed 10 mm from, and perpendicular to, the adaxial surface of the leaves. For each seagrass species, an average value of ΔF/Fm’ was calculated based on measurements made on three young fully expanded mature leaves every two hours from 06:00 to 18:00. Average measurements of Fv/Fm were made in a similar way every day at 05:00 (in darkness, before sunrise). The electron transport rate (ETR) at each light intensity was estimated by multiplying the effective quantum yield (ΔF/Fm’) by the photosynthetic photon flux density (PPFD) received by the leaf, by 0.5 (assuming equal distribution of absorbed photons between PSI and PSII), and by a leaf absorption factor (AF). The absorption factors were determined by measuring the incident irradiance from a LED light source before and after the optic fiber (Diving PAM; Walz) was covered with the seagrass leaves. The AF of each leaf was calculated from the proportion of irradiance absorbed by the leaf in each species (Beer & Björk, 2000). In this study, the average AF (recorded from eight leaves) was 0.658 ± 0.001 (mean ± *SE*) for *C. serrulata*, 0.666 ± 0.001 for *E. acoroides*, 0.676 ± 0.002 for *T. ciliatum*, and 0.730 ± 0.001 for *T. hemprichii*.

### 2.5 Determination of temperature effects on biomass

Aboveground (sheaths and leaves) and belowground (roots and rhizomes) biomass samples were harvested from 0.25 × 0.25 m seagrass sods for each species: (1) in situ (three sods collected at the same time as those used for the mesocosm), and (2) in the experimental containers at the end of the experiment. Plant biomass samples were separated into above‐ and belowground biomass, quickly rinsed, and oven‐dried at 60°C for 24 hr to constant weight. The dry weight of the samples was used to estimate the percentage loss of above‐ and belowground biomass as indicated below:

(1) %lossofbiomass=(B−A)/B×100

where *B* is the weight of biomass before the experiment (i.e., average weight of biomass from in situ estimations) and *A* is the weight of biomass at the end of the experiment. All biomass estimations were based on data from three repeated experiments, as data from the last experiment were lost due to logistical failure.

### 2.6 Data analysis

The effects of temperature on ETR and Fv/Fm were analyzed using repeated‐measures analysis of variance (ANOVA), whereas the effects of temperature on above‐ and belowground seagrass biomass were analyzed using one‐way ANOVA. The analyses were performed separately for each species to be able to assess species‐specific threshold levels of photosynthetic performance and biomass. All main tests were significant, and thus, Tukey's HSD post hoc test was used to determine significant differences between temperature treatments. Homogeneity of variance was tested using Levene's test showing no heterogeneity; hence, all analyses were performed on raw data. *T*‐tests were used to compare % biomass loss between ambient and elevated temperature treatments. All data analyses were performed using Statistica v. 13.

## 3 RESULTS

### 3.1 In situ variations in temperature and light

There was a considerable fluctuation in daily water temperature and light in the seagrass meadows from where the samples were collected (Figure 1). During the study period, the water temperature ranged from 25.6°C to 44.3°C (Figure 1), with 65% of daily maxima exceeding 35°C and 23% even exceeding 40°C. The maximum daily light level during the spring low tide period (i.e., from the 27th of February to the 6th of March), with water depths <0.25 m, was generally between 815 and 2,141 μmol photons m^−^² s^−^¹, and clearly higher than during the rest of the sampling period (Figure 1), when tides were higher during daytime. The experimental conditions (as shown in Figure 2) thus mimic the in situ conditions during spring low tides.

### 3.2 Effects of temperature on electron transport rate (ETR) and maximal quantum yield (Fv/Fm)

**Figure 3:**
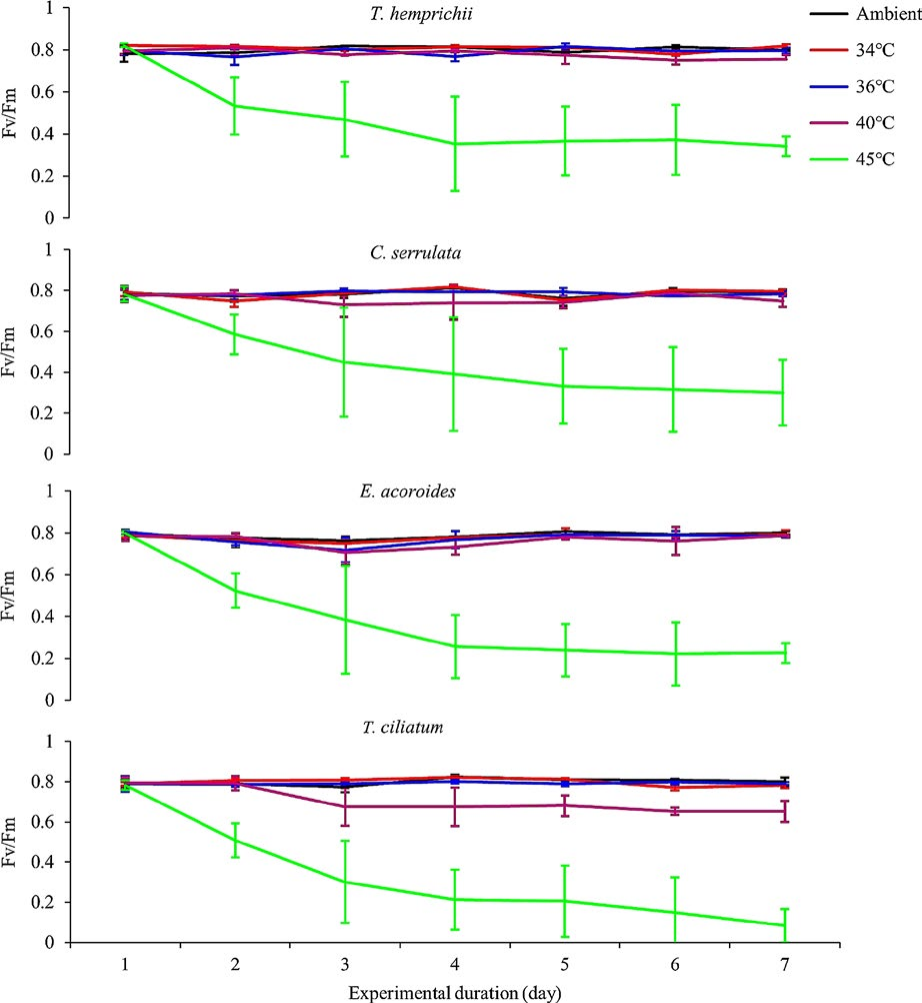
Maximal quantum yield (Fv/Fm) measured in darkness (before sunrise) in four seagrass species exposed to midday temperature stress and ambient light conditions. Bars are means ± *SE* (*n* = 4)

ETR was significantly reduced in all species in the 45°C treatment (repeated‐measures ANOVA, *p *<* *.001; Figure 2), as well as in *T. ciliatum* in the 40°C treatment (repeated‐measures ANOVA, *p *<* *.001; Figure 2). Time (days) had a clear negative effect on ETR in *T. ciliatum* (Figure 2; repeated‐measures ANOVA, *p *<* *.05). No significant effects on ETR were found in any other treatment or species. Similar to ETR, the Fv/Fm of the seagrass leaves measured at 05:00 (before sunrise) was significantly affected only in the 45°C treatment in all species, as well as in the 40°C treatment in *T. ciliatum* (repeated‐measures ANOVA, *p *<* *.001; Figure 3). Time had an effect on Fv/Fm in all species in the 45°C treatment (repeated‐measures ANOVA, *p *<* *.05), while temperature had no effects on Fv/Fm in any of the other treatments.

### 3.3 Effects of temperature on above‐ and belowground seagrass biomass

**Figure 4:**
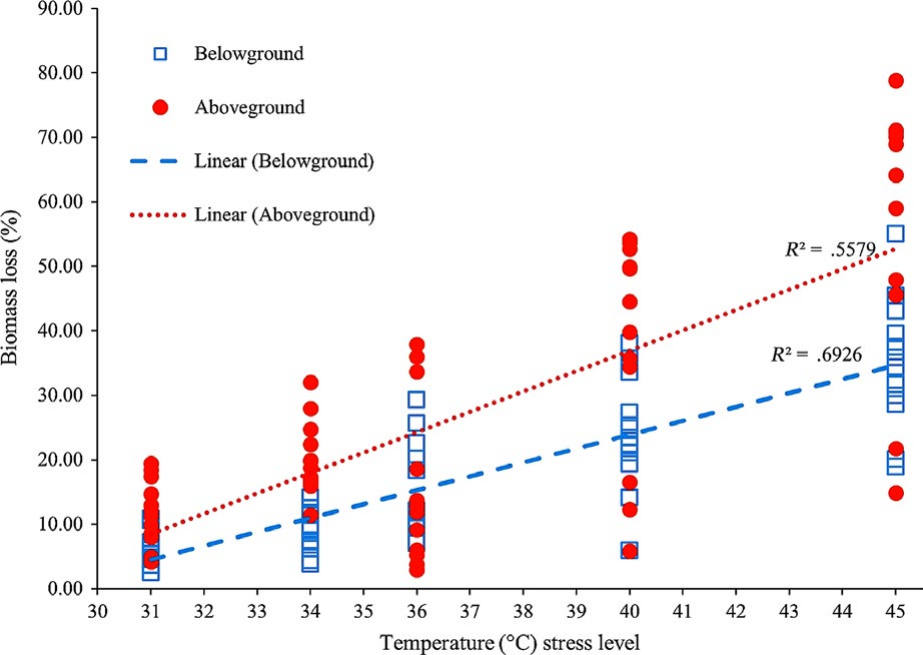
Relationships between temperature stress level and seagrass biomass loss (separated into above‐ and belowground parts), including all data points of three repeated experiments. Dotted lines show significant correlations (*p* < .05) between temperature and above‐ and belowground biomass, respectively. Ambient temperature level (29–33°C) is indicated at its average value of 31°C

**Figure 5:**
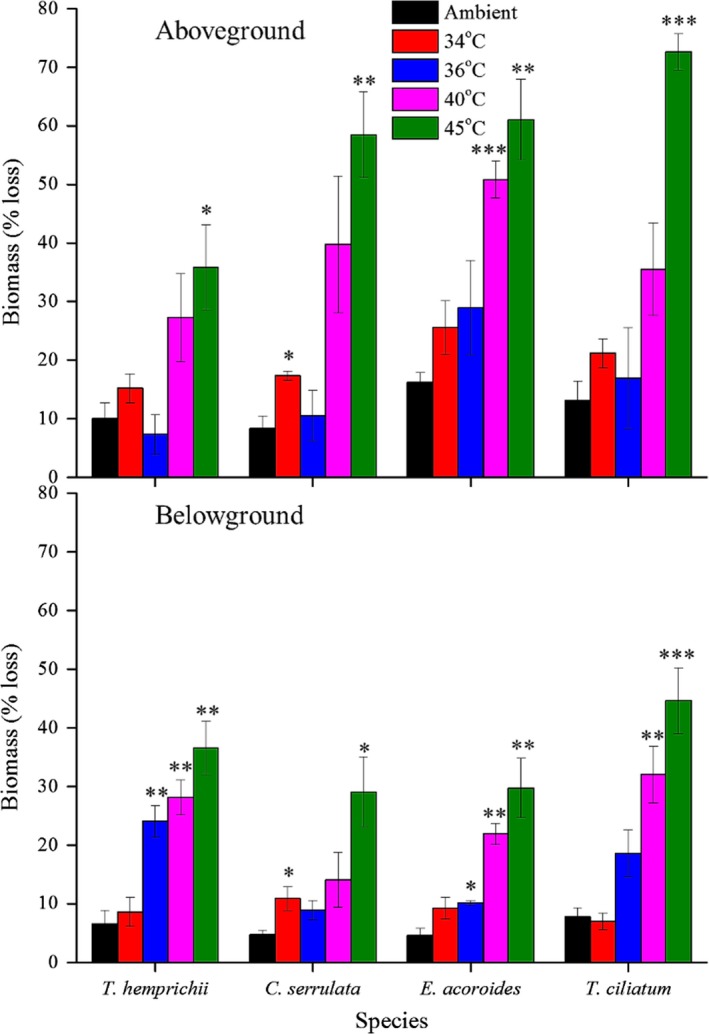
Above‐ and belowground biomass loss of four seagrass species exposed to midday temperature stress and ambient light conditions. Bars show means ± *SE* (*n* = 3). Asterisks indicate significant differences among temperature treatments for each seagrass species separately (*p < .05, **p < .01, and ***p < .001)

**Figure 6:**
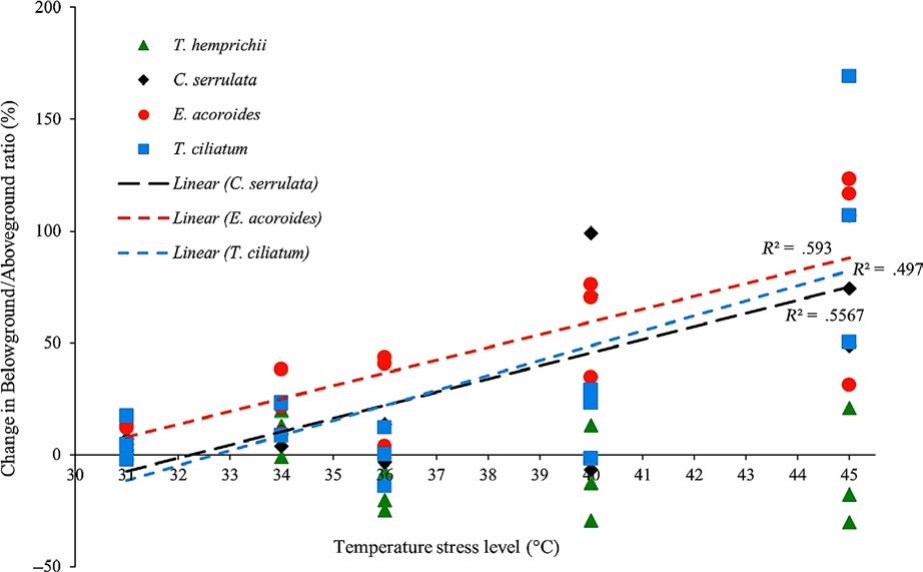
Species’ trends of the change in below‐/aboveground ratio across levels of temperature stress, including all data points of the three repeated experiments. Dotted lines show significant correlations (*p* < .05) between temperature and final below‐/aboveground ratio. Ambient temperature level (29–33°C) is indicated at its average value of 31°C

Generally, we found some common trends in biomass changes for all species across temperature stress levels (Figures 4, 5, 6). Above‐ and belowground seagrass biomass decreased in all treatments (Figures 4 and 5), and there was a significant increase and higher variability in biomass loss with increased temperature stress (Figure 4). A large loss of above‐ground (36%–73% loss) and belowground (29%–45% loss) seagrass biomass was observed in all species in the 45°C treatment (Tukey's HSD tests, *p *<* *.05 and 0.001, respectively; Figure 5). At this temperature stress level, the greatest biomass loss occurred in *T. ciliatum*, in which more than two‐thirds of the aboveground and almost half of the belowground tissue were lost, and with the three other species slightly less, but still highly affected (Figure 5). Aboveground biomass was also significantly reduced in the 40°C treatment of *E. acoroides* (61% loss; Tukey's HSD test, *p *<* *.05) and in the 34°C treatment of *C. serrulata* (17% loss; Tukey's HSD test, *p *<* *.001; Figure 5). In addition, for the belowground counterpart, we found significant biomass reductions in the 40°C treatments of *T. hemprichii*,* E. acoroides*, and *T. ciliatum* (28%, 22% and 32% loss, respectively; Tukey's HSD tests, *p *<* *.01) as well as in the 36°C treatments of *T. hemprichii* (24% loss; Tukey's HSD test, *p *<* *.01) and *E. acoroides* (10% loss; Tukey's HSD test, *p *<* *.05) and the 34°C treatment of *C. serrulata* (9% loss; Tukey's HSD test, *p *<* *.05; Figure 5). The balance between the above‐ and belowground seagrass tissues also changed with temperature. In *C. serrulata*,* E. acoroides*, and *T. ciliatum*, there was a higher proportion of biomass reduction of the aboveground tissue compared to the belowground counterpart (Figure 4), with a clear linear increase in the below‐ to aboveground biomass ratio (*B*/*A* ratio) when the temperature of stress increased (Figure 6). In *T. hemprichii*, there was no clear pattern seen for *B*/*A* ratio (Figure 6).

## 4 DISCUSSION

This study showed that tropical seagrasses are tolerant to repeatedly occurring high temperature stress, and revealed clear differences in how seagrass plants respond depending on if measured as photosynthetic capacity or percent plant‐tissue reduction. Significant effects of recurrent high midday temperature stress were generally found at lower temperatures for biomass (primarily seen in the belowground parts) than for photosynthetic activity, demonstrating that tropical seagrass plants could maintain an apparently unaffected photosynthetic capacity during high temperature stress, while at the same time suffering major losses of biomass. Our results showed a distinct and general threshold level between 40 and 45°C, where the photosynthetic performance was significantly impacted in all species, except in *T. ciliatum*, where the threshold level was between 36 and 40°C. This higher sensitivity seen in *T. ciliatum* might be because this species often grows deeper than the other three seagrasses in the WIO region (Aleem, 1984; Gullström et al., 2002). Where negative effects of temperature were observed on photosynthetic performance, this was exacerbated by the number of days of repeated stress. This clearly indicates a persistent damage in the photosynthetic apparatus, a chronic photoinhibition (Beer, Björk, & Beardall, 2014), as the maximal photosynthetic yield (Fv/Fm) did not recover even after several hours of darkness and ambient temperatures. The decrease in Fv/Fm progressed with time (as also seen for the ETR in *T. ciliatum*), suggesting an additive stress effect. In contrast to the effects on the photosynthetic apparatus, we could not find any distinct threshold level for the negative effects on biomass, which seemed to gradually increasing with temperature.

The primary effect of temperature on biomass seen in our study was a reduction in leaves as the seagrasses (except for *T. hemprichii*) generally lost a higher proportion of the aboveground biomass than of the belowground biomass. This was reflected in the below‐ to aboveground biomass ratio that was positively correlated with temperature and thus the higher temperatures resulted in a stronger effect on the aboveground parts of the plants. These biomass losses may be linked to changes in the net photosynthetic oxygen production in the seagrasses. The loss of photosynthetic activity, and a subsequent reduction in the internal O_2_ transport to nonphotosynthetic parts of the plant, has been shown to cause anoxia in the basal meristematic regions of seagrass leaves (Greve, Borum, & Pedersen, 2003; Nagel, 2007). This response, possibly strengthened by hydrogen peroxide (H_2_O_2_) formation observed in anoxic plant tissue (Blokhina, Virolainen, & Fagerstedt, 2003), could be linked to degradation of the leaf meristems (Greve et al., 2003), which has been observed under events of seagrass die‐off (Borum, Sand‐Jensen, Binzer, Pedersen, & Greve, 2007; Greve et al., 2003; Koch & Erskine, 2001; Koch, Schopmeyer, Kyhn‐Hansen, & Madden, 2007; Nagel, 2007; Pringault, Duran, Jacquet, & Torréton, 2008). In our study, however, significant biomass loss (as compared to the controls) occurred at temperatures where the photosynthetic activity was still unaffected. Thus, it appears that the biomass loss could not only be the result of a reduction in photosynthetic activity and that additional explanations have to be sought. An alternative explanation to the observed effects on biomass could be temperature‐dependent effects on biogeochemical processes within the sediment (surrounding the roots and rhizomes). Increased temperatures will have an increasing effect on the respiratory rates of microorganisms within the sediment (as well as on roots and rhizomes), further lowering oxygen levels in the surrounding sediments (Arnosti, Jørgensen, Sagemann, & Thamdrup, 1998; Kristensen, Bodenbender, Jensen, Rennenberg, & Jensen, 2000; Pringault et al., 2008; Sanz‐Lázaro, Valdemarsen, Marín, & Holmer, 2011; Thamdrup, Hansen, & Jørgensen, 1998), promoting sulfate reducing bacteria possibly resulting in toxic levels of sulfide that could cause both shoot and root–rhizome necrosis (Collier & Waycott, 2014; Holmer et al., 2009; Koch & Erskine, 2001; Lee & Dunton, 2000; Pedersen, Binzer, & Borum, 2004). Such toxic effects of sulfide have also been demonstrated to strengthen the negative effects of temperature stress in *Thalassia testudinum* (García, Holmer, Duarte, & Marbà, 2013; Koch & Erskine, 2001; Koch et al., 2007), and the combination of anoxic conditions and high sulfide levels has been shown to impair growth and enhance seagrass mortality (Holmer et al., 2009; Koch & Erskine, 2001; Koch et al., 2007).

Additionally, it is possible that in our experiment, the seagrasses did experience a reduction in photosynthetically produced oxygen even at temperatures below those that caused decreases in photosynthetic rate. This could have resulted from temperature effects on processes other than the photosynthetic reactions. Firstly, mitochondrial respiration could have consumed proportionally more oxygen at higher temperatures (Atkin & Tjoelker, 2003), and secondly, other oxygen‐consuming processes are known to be enhanced at higher temperatures (Pedersen et al., 2016). It is possible that photorespiration or the Mehler reaction was competing for the reducing power from the electron transport chain (Asada, 1999). In both cases, the ETR would keep on running, but oxygen will be spent in the process, causing a reduction in the net O_2_ production from photosynthesis (Beer et al., 2014). The Mehler reaction has been shown to be of minor importance in the only seagrass that has been investigated (Z*ostera marina*, Buapet & Björk, 2016), but the activity of the photorespiratory pathway can be substantial at conditions with high oxygen and low CO_2_ levels (Buapet & Björk, 2016; Buapet, Rasmusson, Gullström, & Björk, 2013). Such a photorespiratory reduction in net oxygen production may gradually enhance as the water temperature increases, as the oxygenase activity of Rubisco is thought to be increasing with temperature (Sage & Kubien, 2007). Thus, it is possible that, even though the plants had a mostly stable ETR up to the 40°C level, the oxygen transport to the nonphotosynthetic parts of the plants decreased with increasing temperatures. This, possibly together with reduced oxygen content due to reduced oxygen solubility (Truesdale, Downing, & Lowden, 1955), could have caused anoxia in the basal meristematic regions of the leaves by an impaired supply of photosynthetically produced oxygen, with subsequent necrosis causing tissue degradation and leaf detachment. Such a process could have been (at least partly) responsible for the biomass losses seen in this study. It is, however, likely that the biomass effect observed in this study is a consequence of a combined effect of temperature on both the internal physiology of the seagrass plants, and sediment processes within the rhizosphere. The organic carbon content of the selected study site is relatively low, averaging around 0.5% (Gullström et al., 2017), which would by itself not cause harmfully low oxygen levels. The seagrass die‐back seen in this study would, however, increase the oxygen consumption in the sediment while the belowground material is degraded.

Under projected climate change conditions, increases in the intensity and frequency of extreme water temperatures are expected, enhancing the vulnerability of seagrass meadows to temperature‐associated stress (Marba & Duarte, 2010; Pachauri et al., 2014). Our findings demonstrate that extensive losses in plant biomass might occur when affected by prolonged periods of thermal stress, where the water reaches 40°C or higher during shorter thermal spikes.

When assessing effects of such temperature increases, it is important to emphasize both that the response to stress might vary with the parameter used to evaluate the stress (in this study ETR vs. biomass loss), and that the response will to a certain extent be species‐specific. In our study, three of four species showed similar responses to stress, with the morphologically most separate species, *T. ciliatum*, responding in a different way, that is, by displaying a more sensitive photosynthetic activity. Also other studies have shown differences that might be coupled to differences in morphology or distribution; for instance, the small, cosmopolitan *Halophila ovalis*, with a belowground part distributed mostly on the top of the sediment, has been found more sensitive to high spikes in temperatures compared to larger species such as *Cymodocea rotundata*,* Halodule uninervis*,* Thalassia hemprichii*, and *Cymodocea serrulata* (Campbell et al., 2006; Collier & Waycott, 2014).

At a larger scale perspective, the loss of seagrass biomass from coastal waters would affect a range of ecosystem services (Cullen‐Unsworth et al., 2014; Orth et al., 2006) such as sediment stabilization (Newell & Koch, 2004), the nursery habitat function (Heck, Hays, & Orth, 2003), and fisheries productivity (Nordlund, Unsworth, Gullström, & Cullen‐Unsworth, 2017). As seagrass beds are considered a major sink for atmospheric CO_2_ (Kennedy & Björk, 2009), such loss of seagrass cover would decrease the carbon sequestration capacity of coastal seas (Dahl et al., 2016; Deyanova et al., 2017), eventually resulting in a decrease in the long‐term carbon storage (Mcleod et al., 2011). Furthermore, a loss and decay of biomass, especially the belowground parts, has also been suggested to promote the production of sulfide and methane gas emissions from the affected meadow (Lyimo et al., 2017). Thus, the effects of increased temperature stress (caused by the greenhouse effect) would result in a weakening in the blue carbon sink function of coastal waters, and also the increased emission of greenhouse gases.

## CONFLICT OF INTEREST

None declared.

## AUTHOR CONTRIBUTIONS

Rushingisha George performed conception and design of work, data collection, analysis and interpretation of data. Martin Gullström and Mats Björk performed conception and design of work, drafting the work, data analysis and revising critically for intellectual content. Mwita M. Mangora and Matern S.P. Mtolera performed review, drafting, and final approval of the version to be submitted.
