# Supplementary material for: High midday temperature stress has stronger effects on biomass than on photosynthesis: A mesocosm experiment on four tropical seagrass species
Source: Ecol Evol. 2018 Apr 10;8(9):4508–17. doi: 10.1002/ece3.3952 (PMC5938440; doi:10.1002/ece3.3952)
Supplement: Supplementary file 1 [file ECE3-8-4508-s001.docx]

**SUPPLEMENTARY TABLE 1** Seagrass biomass data

| **Species** | **Temperature stress level** | **BG Biomass (g DW m^-2^)** | | **AG Biomass (g DW m^-2^)** | | **Total biomass (BG+AG), g DW m^-2^)** | | **BG/AG ratio** | |
| --- | --- | --- | --- | --- | --- | --- | --- | --- | --- |
|  |  | **Initial** | **Final** | **Initial** | **Final** | **Initial** | **Final** | **Initial** | **Final** |
| ***Thalassia hemprichii*** | **Ambient** | **765.71 ± 39.28** | **713.39 ± 20.45** | **414.13 ± 54.35** | **371.41 ± 39.92** | **1179.84 ± 79.5** | **1084.8 ± 45.31** | **1.91 ± 0.25** | **1.97 ± 0.23** |
|  | **34ºC** | **765.71 ± 39.28** | **699.47 ± 38.26** | **414.13 ± 54.35** | **344.96 ± 51.29** | **1179.84 ± 79.5** | **1044.43 ± 54.88** | **1.91 ± 0.25** | **2.14 ± 0.39** |
|  | **36ºC** | **765.71 ± 39.28** | **579.68 ± 8.93** | **414.13 ± 54.35** | **380.32 ± 43.2** | **1179.84 ± 79.5** | **960.00 ± 41.16** | **1.91 ± 0.25** | **1.56 ± 0.18** |
|  | **40ºC** | **765.71 ± 39.28** | **550.67 ± 36.87** | **414.13 ± 54.35** | **333.12 ± 39.07** | **1179.84 ± 79.5** | **883.79 ± 56.83** | **1.91 ± 0.25** | **1.69 ± 0.20** |
|  | **45ºC** | **765.71 ± 39.28** | **486.88 ± 48.15** | **414.13 ± 54.35** | **293.71 ± 36.46** | **1179.84 ± 79.5** | **780.59 ± 14.57** | **1.91 ± 0.25** | **1.74 ± 0.34** |
| ***Cymodocea serrulata*** | **Ambient** | **309.81 ± 40.47** | **295.36 ± 39.37** | **169.55 ± 24.24** | **156.37 ± 25.72** | **479.36 ± 64.57** | **451.73 ± 65.01** | **1.84 ± 0.05** | **1.91 ± 0.08** |
|  | **34ºC** | **309.81 ± 40.47** | **277.23 ± 41.79** | **169.55 ± 24.24** | **140.53 ± 21.42** | **479.36 ± 64.57** | **417.7 6± 62.81** | **1.84 ± 0.05** | **1.98 ± 0.08** |
|  | **36ºC** | **309.81 ± 40.47** | **280.96 ± 31.74** | **169.55 ± 24.24** | **152.91 ± 25.69** | **479.36 ± 64.57** | **433.87 ± 56.88** | **1.84 ± 0.05** | **1.88 ± 0.16** |
|  | **40ºC** | **309.81 ± 40.47** | **269.92 ± 49.77** | **169.55 ± 24.24** | **97.33 ± 7.55** | **479.36 ± 64.57** | **367.25 ± 49.62** | **1.84 ± 0.05** | **2.81 ± 0.53** |
|  | **45ºC** | **309.81 ± 40.47** | **218.56 ± 29.89** | **169.55 ± 24.24** | **68.8 ± 11.31** | **479.36 ± 64.57** | **287.36 ± 40.71** | **1.84 ± 0.05** | **3.22 ± 0.22** |
| ***Enhalus acoroides*** | **Ambient** | **1583.15 ± 95.56** | **1511.68 ± 110.01** | **616.37 ± 50.52** | **517.07 ± 49.42** | **2199.52 ± 142.97** | **2028.75 ± 158.43** | **2.58 ± 0.01** | **2.94 ± 0.10** |
|  | **34ºC** | **1583.15 ± 95.56** | **1438.08 ± 100.50** | **616.37 ± 50.52** | **460.96 ± 58.80** | **2199.52 ± 142.97** | **1899.04 ± 157.79** | **2.58 ± 0.01** | **3.17 ± 0.18** |
|  | **36ºC** | **1583.15 ± 95.56** | **1421.28 ± 79.74** | **616.37 ± 50.52** | **430.19 ± 12.15** | **2199.52 ± 142.97** | **1851.45 ± 75.31** | **2.58 ± 0.01** | **3.31 ± 0.23** |
|  | **40ºC** | **1583.15 ± 95.56** | **1237.87 ± 92.52** | **616.37 ± 50.52** | **303.79 ± 33.61** | **2199.52 ± 142.97** | **1541.65 ± 105.52** | **2.58 ± 0.01** | **4.16 ± 0.46** |
|  | **45ºC** | **1583.15 ± 95.56** | **1113.44 ± 107.28** | **616.37 ± 50.52** | **245.97 ± 60.79** | **2199.52 ± 142.97** | **1359.41 ± 157.42** | **2.58 ± 0.01** | **4.92 ± 0.80** |
| ***Thalassondron ciliatum*** | **Ambient** | **438.51 ± 43.02** | **405.6 ± 44.93** | **351.73 ± 25.52** | **304.12 ± 13.05** | **790.24 ± 66.37** | **709.71 ± 57.99** | **1.24 ± 0.06** | **1.33 ± 0.10** |
|  | **34ºC** | **438.51 ± 43.02** | **408.85 ± 45.06** | **351.73 ± 25.52** | **276.11 ± 11.69** | **790.24 ± 66.37** | **684.96 ± 54.94** | **1.24 ± 0.06** | **1.47 ± 0.12** |
|  | **36ºC** | **438.51 ± 43.02** | **357.44 ± 42.27** | **351.73 ± 25.52** | **288.12 ± 16.45** | **790.24 ± 66.37** | **645.55 ± 53.55** | **1.24 ± 0.06** | **1.24 ± 0.12** |
|  | **40ºC** | **438.51 ± 43.02** | **294.027 ± 12.37** | **351.73 ± 25.52** | **205.23 ± 15.75** | **790.24 ± 66.37** | **499.25 ± 28.05** | **1.24 ± 0.06** | **1.44 ± 0.05** |
|  | **45ºC** | **438.51 ± 43.02** | **239.47 ± 21.01** | **351.73 ± 25.52** | **95.84 ± 11.80** | **790.24 ± 66.37** | **335.31 ± 10.84** | **1.24 ± 0.06** | **2.64 ± 0.55** |
